# Combined Microbial Consortium Inoculation and Black Locust Planting Is Effective in the Bioremediation of Waste Drill Cuttings

**DOI:** 10.3389/fmicb.2020.536787

**Published:** 2020-09-30

**Authors:** Hanjun Liu, Lirong Chen, En T. Wang, Yihao Liu, Lingzi Zhang, Ke Zhao, Yunfu Gu, Xiumei Yu, Menggen Ma, Petri Penttinen, Xiaoping Zhang, Min Huang, Liangji Deng, Qiang Chen

**Affiliations:** ^1^College of Resource Sciences and Technology, Sichuan Agricultural University, Chengdu, China; ^2^Safety and Environmental Protection Quality Supervision and Testing Research Institute, CNPC Chuanqing Drilling Engineering Co. Ltd., Guanghan, China; ^3^Departamento de Microbiologia, Escuela Nacional de Ciencias Biologicas, Instituto Politecnico Nacional, Mexico City, Mexico

**Keywords:** waste drill cutting, bioaugmentation plus phytoremediation, enzyme activity, microbial communities, partial least squares path modeling

## Abstract

Waste drill cuttings (WDCs), produced during gas and oil drilling consisting of 80% rock cuttings and 20% drilling muds, are an increasingly potent source of environmental pollution. We studied the efficiency of bioaugmentation and phytoremediation to remediate WDCs in an experiment where WDCs were incubated in a greenhouse for 120 days with and without black locust (*Robinia pseudoacacia*) plant and with or without bacterial and fungal consortium inoculant. The pollutant removal rates were highest in inoculated and planted treatment, followed by inoculated treatment and planted treatment. The small decrease in contaminant level in the control treatment suggested that indigenous microorganisms in WDCs had little pollutant degradation capability. In the inoculated and planted treatments, after 120 days, the germination rate of red clover seeds was on the same level as in the natural soil, showing a marked decrease in the ecotoxicity of WDC. Both the bacterial and fungal richness and bacterial diversity increased in all the treatments over time, whereas fungal diversity increased only in the not-inoculated treatments. The activity of laccase enzyme played a key role in the bioremediation process. The enzyme activities were mostly governed by inoculated consortium and soil bacterial community, and black locust affected the bioremediation mainly through its effect on N content that further affected bacterial and fungal communities.

## Highlights

- Waste drill cuttings (WDCs) are a potent source of environmental pollution.
- Bioremediation of WDCs with a combination of bioaugmentation and phytoremediation is an attractive strategy.

- Lignin degradation enzyme activities were the primary factors correlated to the contaminant removal in WDC bioremediation.
- Enzyme activities were mostly governed by inoculated consortium and soil bacterial community.
- Black locust affected the bioremediation through N content that affected bacterial and fungal communities.

## Introduction

Waste drill cutting (WDC) produced during gas and oil drilling consists of 80% rock cuttings and 20% drilling muds. Drilling muds are applied to lubricate and cool the drill bit, maintain hydrostatic equilibrium, and move drill cuttings to the surface during gas and oil drilling (Okoro, 2011; Fattah and Lashin, 2016). To achieve desirable rheological properties and density, drilling mud is amended with additives, for example, with a liquid (water or diesel, mineral or synthetic oil), a weighting agent (barium or calcium sulfate), amargosite, sulfonated phenol formaldehyde (SPF) resin, and sulfonated lignite (SL) (Fink, 2012). However, using oils, SPF resin and SL create potential environmental hazards, for example, since these additives are ecotoxic and increase the chemical oxygen demand (COD). Approximately 2.5 million tons of WDCs are produced annually in China (Sun et al., 2016), making WDC an increasingly potent source of environmental pollution.

Among the remediation methods, bioremediation is considered an efficient, low-cost technology to treat polluted soils and sediments (Mutairi et al., 2008; Cerqueira et al., 2014). Bioremediation methods may be divided into five types—phytoremediation, biostimulation, bioaugmentation, natural attenuation, and bioventing—out of which the first three are most commonly used. Bioaugmentation involves inoculating exogenous contaminant-degrading microbes. In biostimulation, nutrients are added to stimulate the indigenous community and to avoid metabolic limitations. Phytoremediation involves the utilization of plants to extract, accumulate, degrade, filter, stabilize, and volatilize contaminants. Biostimulation, bioaugmentation, and phytoremediation approaches can be used in combination. The inoculated bacteria compete with the bacteria already present, and a successful establishment of the inoculum is required for long-term efficiency (Gkorezis et al., 2016). Vegetated soils are capable of supporting high microbial numbers and diversity, thus combining bioaugmentation with plants that provide nutrients for bacterial growth may affect the establishment of the inoculum (Gkorezis et al., 2016). Understanding how bioremediation affects the populations of pollutant-degrading microbes, the diversity and activity of the microbial community and the adaptability of exogenous microbes into the contaminated environment are essential in ensuring effective bioremediation (Kaplan and Kitts, 2004; Kauppi et al., 2011; Liu et al., 2012; Taccari et al., 2012). Bioaugmentation and biostimulation have been used to remediate WDC and waste drilling fluids (Rojas-Avelizapa et al., 2007; Chen et al., 2015; Avdalovic et al., 2016; Zha et al., 2017). To our knowledge, combined phytoremediation and bioaugmentation in WDC bioremediation has not been studied to date, and the relative contribution of changes in microbial communities to WDC bioremediation efficiency has not received attention. The objectives of this research were (1) to evaluate the efficiency of combined bioaugmentation and phytoremediation in organic pollutant degradation using a fungal and bacterial consortium as microbial inoculant and *Robinia pseudoacacia* (black locust), (2) to determine changes in microbial communities during the WDC bioremediation, and (3) to estimate the relative contributions of treatments and microbial communities on WDC bioremediation efficiency.

## Materials and Methods

### Soil Collection and Pretreatment

Water-based WDCs, produced in drilling gas wells, were collected from a WDC centralized treatment point in Deyang, China (E, 31°16′; N, 104°11′). Natural soil (NS) was collected from an agricultural field in Chengdu, China (E, 31°6′; N, 102°59′). The WDCs were loose and of dark gray color. Before the experiment, NS and WDC were dried and passed through a 2-mm sieve. The physicochemical properties of NS and WDC are in Supplementary Table S1.

### Experimental Setup

The fungal consortium consisted of *Pseudallescheria ellipsoidea* WNF-15 (accession number MG976626), *Stachybotrys chartarum* WNF-20 (MG976627), and *Scopulariopsis brevicaulis* WNF-22 (MG976628). The three strains, with the ability to degrade drilling mud additives such as sulfonated lignite and sulfonated phenolic resin, were isolated and identified in a previous study (unpublished). The strains were cultivated separately in PDA liquid medium at 25°C (Sambrock and Russel, 2001) for 5 days, then inoculated onto a distilled solid medium (80% wheat bran, 20% rice bran, pH 7.0) at the ratio of 3% (v/w), and incubated at 25°C in the dark for 5–7 days. After a large amount of spores formed, equal quantities of the strains were mixed as the fungal inoculum with 3.6 × 10^7^ CFU g^–1^.

The bacterial consortium consisted of *Sinobaca* sp. SCAU3 (accession number KP241934), *Belliella pelovolcani* JH3 (KX230135), *Halomonas* sp. JH4 (KX230136), and *Bacillus halodurans* JU5 (KX230139). The four strains are capable of degrading diesel and decreasing COD in waste drilling mud (Leng, 2013). The strains were cultivated in LB broth at 28°C (Sambrock and Russel, 2001) for 36 h. Cells were collected by centrifugation and resuspended in sterile water to gain an optical density of 1.0 at 600 nm, mixed in equal volumes, and used as the bacterial inoculum with 4.8 × 10^7^ CFU ml^–1^.

Experiments were carried out in 4-L plastic [polyvinyl chloride (PVC)] pots (top diameter 40.6 cm, bottom diameter 14.4 cm, height 44.5 cm) with 4.5 kg WDC per pot. The experimental treatments were (1) WDC: WDC without inoculum or plant; (2) WDC + M: WDC inoculated with bacterial consortium in suspension (0.5% v/w) and solid fungal consortium (0.5% w/w); (3) WDC + P: WDC planted with one 1-year-old black locust (*R. pseudoacacia*) sapling per pot; and (4) WDC + M + P: WDC inoculated with the bacterial consortium in suspension (0.5% v/w) and solid fungal consortium (0.5% w/w) and planted with black locust. Each treatment was carried out in triplicate.

The pots were incubated in a greenhouse under simulated natural illumination conditions, the temperature was 21°C at day and 15°C at night, with a 16-h photoperiod. Soil moisture of each treatment was kept at 60% maximum water holding capacity by adding distilled water. The experiment was started in December 1, 2017.

Soil samples were collected at 0, 15, 30, 60, 90, and 120 days. Two hundred grams of the fresh sample was air-dried and sieved (0.25 mm) for determining physicochemical properties, 50 g was stored at 4°C for enzyme assays within 1 week, and 10 g was stored at −70°C for the subsequent microbial community analyses.

### Total Nitrogen Analysis

Total nitrogen (TN) was analyzed by the semi-micro Kjeldahl method (Bremner, 1996), where 1 g of soil sample was digested with 1.1 g of K_2_SO_4_:CuSO_4_:Se (100:10:1 mass ratio) and 5 ml of H_2_SO_4_. The digestion solution was distilled in a semi-micro Kjeldahl apparatus and titrated with 0.005 M H_2_SO_4_.

### Enzyme Activity Assays

Soil (4.0 g soil wet weight) was mixed with 40 ml of double-distilled water. The solution was incubated for 60 min in a rotary shaker at 120 rpm and centrifuged at 11,000 × *g* for 10 min at 4°C. The supernatant was collected for the enzyme assays.

Laccase (Lac) activity was determined by monitoring the oxidation of 2,2′-azino-bis(3-ethylbenzothiazoline-6-sulfonic acid) (ABTS) at 25°C (Wolfenden and Wilson, 1982) in a 2-ml reaction mixture containing 0.1 ml soil extract, B&R buffer (0.1 M acetic acid, 0.1 M boric acid, 0.1 M phosphoric acid, pH 5.0), and 1 mM ABTS. The absorbance of the solution was determined with a spectrophotometer (model 752, CANY, China), and Lac activity was calculated from the increase in absorbance at 420 nm (ε420 = 36,000 M^–1^cm^–1^). Manganese peroxidase (MnP) activity was determined by monitoring the formation of Mn^3+^-malonate complex in 50 mM sodium malonate buffer (pH 4.5) with 0.5 mM MnSO_4_ at 270 nm (ε = 11,590 M^–1^ cm^–1^) (Wariishi et al., 1992). The reaction was initiated by adding H_2_O_2_ to the final concentration of 0.1 mM. Lignin peroxidase (LiP) activity was determined by monitoring the oxidation of veratryl alcohol to veratraldehyde at 310 nm (ε = 9,300 M^–1^ cm^–1^) in a 1-ml reaction mixture containing 2 mM veratryl alcohol, 50 mM tartaric acid (pH 3), and 0.4 mM H_2_O_2_ (Tanaka et al., 2009). Enzyme absorption spectra were determined at room temperature in a cuvette with a 1-cm light path using spectrophotometer (model 752, CANY, China). One unit of enzymatic activity (U) was defined as the amount of enzyme that transformed 1 μmol of substrate per minute.

### Pollutant Content and Ecotoxicity Analysis

Total petroleum hydrocarbon (TPH) and total organic carbon (TOC) content removal rates and decreases in COD were employed as indicators of bioremediation. The residual TPH was measured using a gravimetric method (Mishra et al., 2001). COD was measured using the rapid digestion spectrophotometry method in a 5B-3C (V8) dry thermostat reactor (Ministry of Ecology and Environment the People’s Republic of China, 2008). TOC was measured using the Walkley–Black method (Sparks et al., 1996).

Germination tests were done using the method of Saterbak et al. (2009). Red clover (*Trifolium pratense*) seeds were sterilized in 0.5% sodium hypochlorite solution for 20 min, then rinsed with sterile distilled water three times, and dried with sterilized filter paper. Fifty seeds were placed onto a 20-g wet weight sample of WDC in a plastic plate. Germination percentage was calculated after incubation at 25°C in the dark for 5 days. COD analysis and germination test included natural soil samples for comparison.

### DNA Extraction and Sequencing

Samples for microbial community analysis were collected at days 0, 60, and 120. At day 0, planted samples were mixed with the respective WDC and WDC + M samples. DNA was extracted from 0.5 g using Fast DNA SPIN Kit (MP Biomedicals, Illkirch, France) following the manufacturer’s instructions. V3–V4 region of the bacterial 16S rRNA gene was amplified using the primers 338F and 806R (Lane, 1991; McBain et al., 2003), and the fungal 18S rRNA gene fragment was amplified using the primers 817F and 1196R (Borneman and Hartin, 2000) as described by Chen et al. (2016). The resulting amplicons were sequenced using Illumina MiSeq reagent kits V3 (600 cycles, MS-102-3003) (PE300 for bacteria, PE250 for fungi) and platform at Personal Biotechnology Co., Ltd., China. Sequences were analyzed using MOTHUR (version 1.34.0) (Schloss et al., 2009). Sequence reads were assigned to each sample according to sample-specific barcodes. Sequences were regarded as low quality and removed if they did not meet the following criteria: exact match to barcode and primers, sequences longer than 200 nucleotides without ambiguous base pairs, and high average quality score (*Q* ≥ 20).

After quality filtering, the 16S rRNA and 18S rRNA gene amplicons were clustered into operational taxonomic units (OTUs) at 97% nucleotide similarity. Taxonomic characterization of the representative sequences of the OTUs was done using the SILVA database (SILVA Release 123). The Simpson and Shannon diversity indexes were calculated using the Mothur program (version 1.34.0) (Schloss et al., 2009). The heatmap was produced using HemI (Heatmap Illustrator, v. 1.0). The sequences have been submitted to the NCBI Sequence Read Archive under accession numbers PRJNA601856 and PRJNA609003.

### Statistical Analysis

Differences in chemical characteristics and enzyme activities between treatments over time were tested using three-way mixed ANOVA with plant and inoculation as the between-subjects factors and time as the within-subject factor, followed by computing simple interactions, simple main effects, and multiple pairwise comparisons in R v.3.6.3 with package rstatix (Kassambara, 2020b; R Core Team, 2020). Differences in COD and seed germination rate between the natural soil and the treatments over time were tested using two-way mixed ANOVA with group as the between-subjects factor and time as the within-subject factor, followed by computing simple main effects in R package rstatix. The results were visualized using R package ggpubr (Kassambara, 2020a). Spearman correlations between the dominant taxa and TOC removal rate were tested using one-way ANOVA and Fisher’s least significant difference (*P* < 0.05) in IBM SPSS Statistics for Windows 20.0 (IBM Corp., Armonk, NY, United States). Differences in microbial community composition based on Bray–Curtis dissimilarities were tested using permutational multivariate analysis of variance (PERMANOVA) and visualized using non-metric multidimensional scaling (NMDS) in PRIMER v7 (Anderson et al., 2008; Clarke and Gorley, 2015). Taxa that characterized the differences between treatments were identified using the linear discriminant analysis (LDA) effect size (LEfSe) method (Segata et al., 2011). The relationships between TN, bacterial and fungal communities, enzyme activities, and organic fraction removal were analyzed using partial least squares path modeling (PLS-PM) according to Tenenhaus et al. (2005) and Ai et al. (2018). The estimates of path coefficients and the coefficients of determination (*R*^2^) in the path model were validated using the package plspm (1,000 bootstraps) in R v.3.3.3 (R Core Team, 2017).

## Results

### Pollutant Removal, Germination Rate, and Nitrogen Content

**FIGURE 1:**
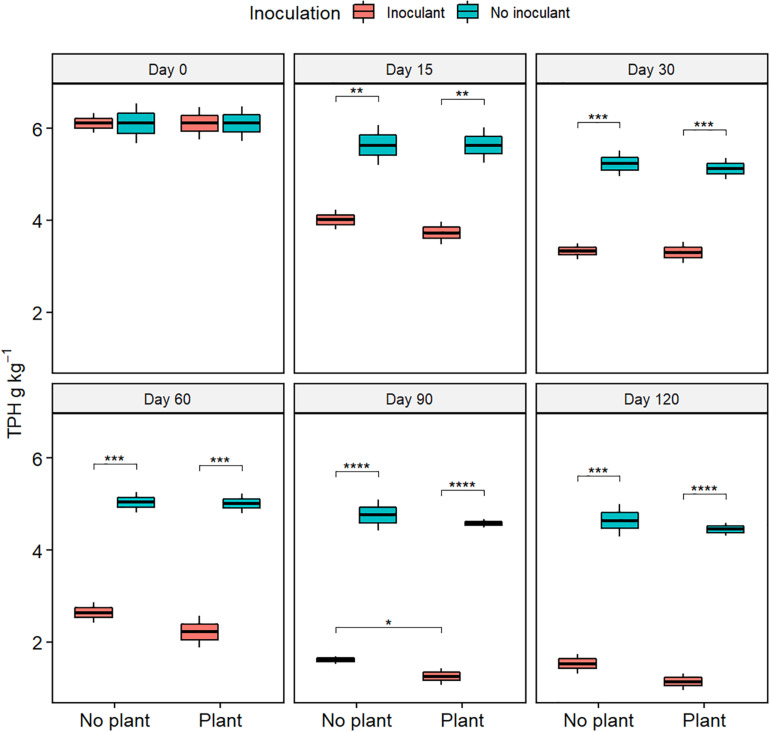
Total petroleum hydrocarbon (TPH) contents of soil extracts from waste drill cuttings (WDCs). WDCs were incubated in a greenhouse for 120 days with and without black locust (*Robinia pseudoacacia*) plant and with or without bacterial and fungal consortium inoculant. Statistically significant differences are indicated with asterisks: *P < 0.05; **P < 0.01; ***P < 0.001; ****P < 0.0001. The boxes show mean, lower and upper hinges indicate the first and third quartiles, and the whiskers indicate the ranges 1.5 times the interquartile range.

The TPH content, TOC content, and the COD decreased with time in all treatments (Figure 1, Supplementary Figures S1, S2, and Supplementary Table S2). At day 120, TPH content was lower in the inoculated treatments WDC + M and WDC + M + P than in the not-inoculated treatments WDC and WDC + P (*P* < 0.05) (Figure 1), suggesting that inoculation with fungal and bacterial consortium enhanced the removal of TPH. Similarly, at day 120, TOC contents and COD were lower in the inoculated treatments than in the not-inoculated treatments (*P* < 0.05) (Supplementary Figures S1, S2). In addition, at day 120, TOC content was slightly lower in the planted and inoculated treatment than in its not planted counterpart (*P* < 0.05) (Supplementary Figure S1), and COD was slightly lower in the planted treatments than in their not planted counterparts (*P* < 0.05) (Supplementary Figure S2). In WDC + M + P, COD was on the same level as in the natural soil (Supplementary Figure S3). Both the TOC and COD decreases suggested that combining black locust with fungal and bacterial consortium inoculation enhanced the degradation of pollutant matter in WDC.

**FIGURE 2:**
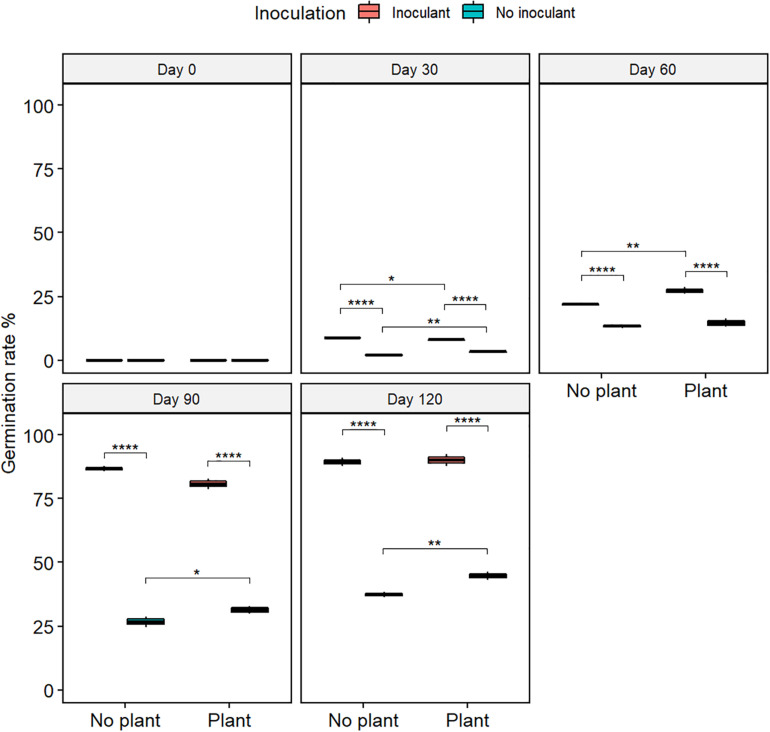
The germination rate of red clover seeds in waste drill cuttings (WDCs). WDCs were incubated in a greenhouse for 120 days with and without black locust (*Robinia pseudoacacia*) plant and with or without bacterial and fungal consortium inoculant. Statistically significant differences are indicated with asterisks: *P < 0.05; **P < 0.01; ****P < 0.0001. The boxes show mean, lower and upper hinges indicate the first and third quartiles, and the whiskers indicate the ranges 1.5 times the interquartile range.

**FIGURE 3:**
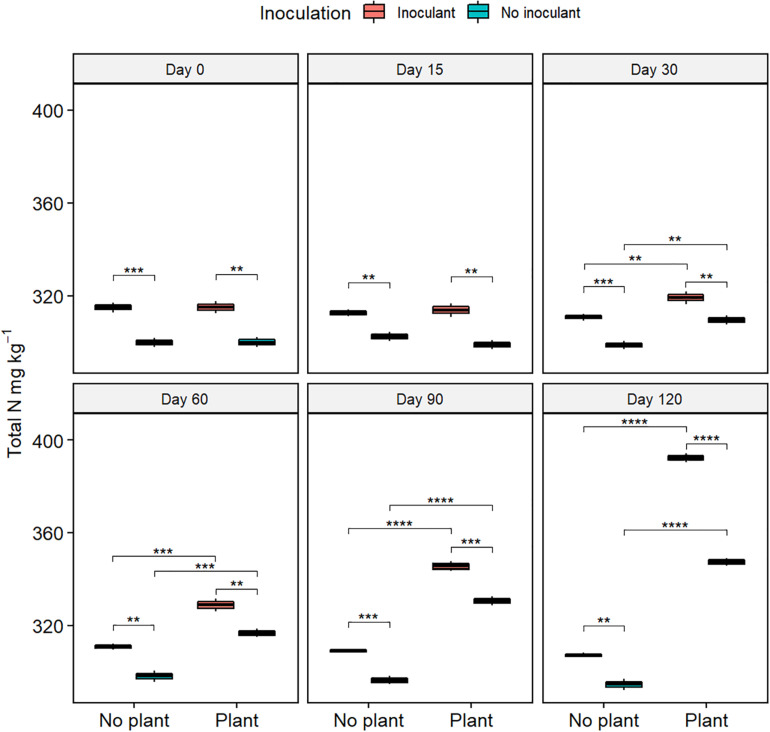
Total nitrogen (TN) content in waste drill cuttings (WDCs). WDCs were incubated in a greenhouse for 120 days with and without black locust (*Robinia pseudoacacia*) plant and with or without bacterial and fungal consortium inoculant. Statistically significant differences are indicated with asterisks: **P < 0.01; ***P < 0.001; ****P < 0.0001. The boxes show mean, lower and upper hinges indicate the first and third quartiles, and the whiskers indicate the ranges 1.5 times the interquartile range.

The germination rate of red clover seeds increased from the initial 0% in all treatments (Figure 2 and Supplementary Table S2), and the total nitrogen (TN) content increased with time in the planted treatments (Figure 3). At day 120, the germination rates in the inoculated treatments were on the same level as in the natural soil and in the not-inoculated treatments approximately half of that in natural soil (*P* ≤ 0.05) (Supplementary Figure S4), suggesting that the ecotoxicity of WDC had markedly decreased due to the inoculation. At day 120, TN content was highest in the inoculated and planted treatment (*P* ≤ 0.05) (Figure 3).

### Enzyme Activity

**FIGURE 4:**
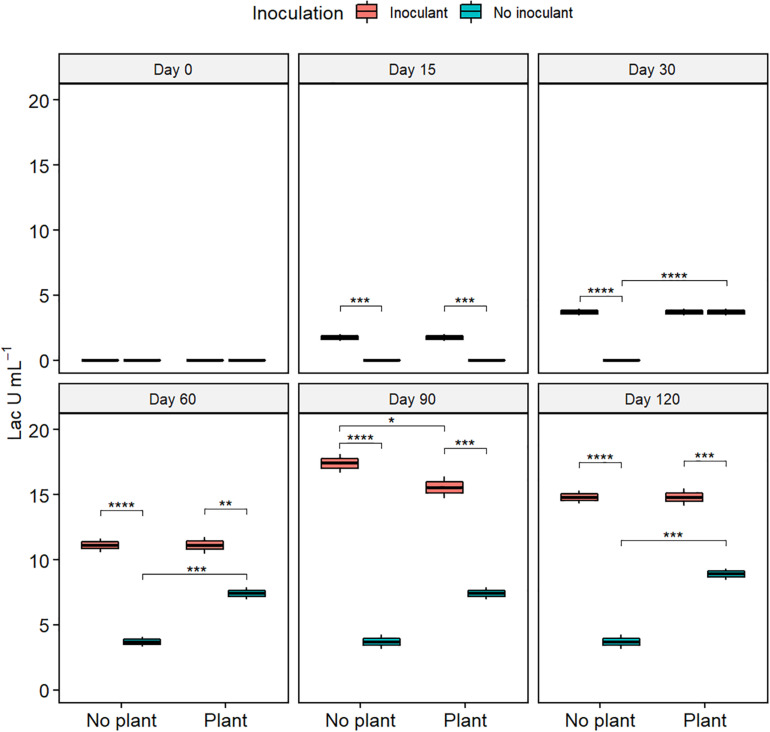
Activity of laccase (Lac) in waste drill cuttings (WDCs). WDCs were incubated in a greenhouse for 120 days with and without black locust (*Robinia pseudoacacia*) plant and with or without bacterial and fungal consortium inoculant. Statistically significant differences are indicated with asterisks: *P < 0.05; **P < 0.01; ***P < 0.001; ****P < 0.0001. The boxes show mean, lower and upper hinges indicate the first and third quartiles, and the whiskers indicate the ranges 1.5 times the interquartile range.

Lac activity increased from the initial 0 in all treatments (Figure 4). At day 120, the Lac activity was highest in the inoculated treatments and lowest in WDC (*P* ≤ 0.05). MnP activity remained nearly unchanged in the not-inoculated treatments during the experiment (Supplementary Figure S5). In the inoculated treatments, MnP activity increased from day 15 to day 60, followed by a sharp decrease. Generally, MnP activity was highest in the WDC + M + P treatment. Changes in LiP activity showed no clear pattern (Supplementary Figure S6).

### Microbial Community

**FIGURE 5:**
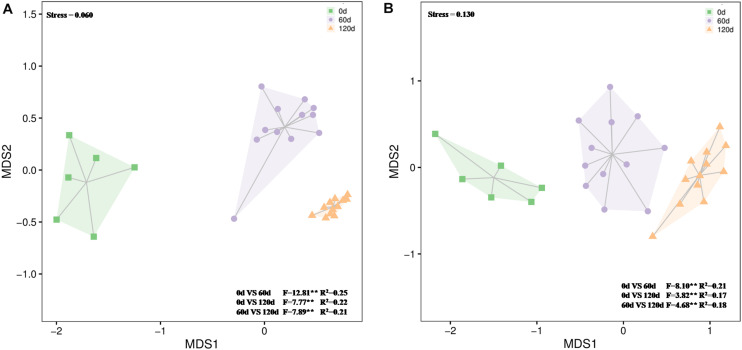
Differences in bacterial **(A)** and fungal **(B)** community composition over time in waste drill cuttings (WDCs). WDCs were incubated in a greenhouse for 120 days with and without black locust (*Robinia pseudoacacia*) plant and with or without bacterial and fungal consortium inoculant. The differences are based on Bray–Curtis dissimilarities and tested using permutational multivariate analysis of variance (PERMANOVA). **P < 0.01.

**FIGURE 6:**
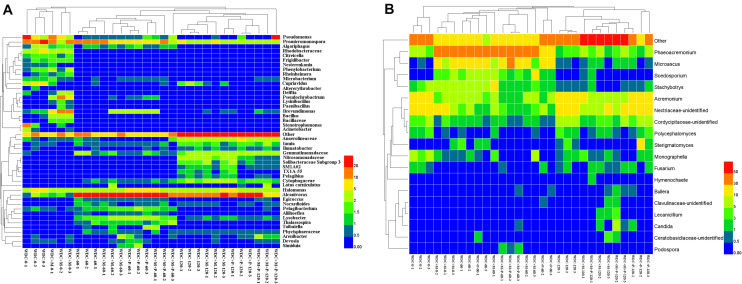
Dominant bacterial **(A)** and fungal **(B)** taxa in waste drill cuttings (WDCs). WDCs were incubated in a greenhouse for 120 days with and without black locust (*Robinia pseudoacacia*) plant and with or without bacterial and fungal consortium inoculant. WDC, no plant and no inoculant; WDC + M, no plant and inoculant; WDC + P, plant and no inoculant; WDC + M + P, plant and inoculant.

The 16S rRNA gene amplicon sequencing resulted in 1,663,757 reads that were classified into 3,062 OTUs. Chao1 richness was lower in the beginning than after 60 and 120 days (*P* ≤ 0.05) (Supplementary Table S3). Shannon diversity and Pielou’s evenness were lowest in the beginning, and in all treatments highest and approximately on the same level at day 120 (*P* ≤ 0.05) (Supplementary Table S3). At the phylum level, the relative abundances of OTUs assigned into Proteobacteria, Actinobacteria, Chloroflexi, Bacteroidetes, and Gemmatimonadetes were high in all treatments, and communities at day 120 were distinct from those at days 0 and 60 (Figure 5A and Supplementary Figure S7). Based on the most abundant taxa, the communities clustered mostly based on time and less based on treatments (Figure 6A).

We identified the taxa characterizing the bacterial communities in different treatments using the LEfSe method. Most of the taxa with a large effect size (LDA score > 4.0) were associated with the initial stages (Supplementary Figure S7 and Supplementary Table S4). The relative abundance of *Promicromonospora* was high in WDC and WDC + M at day 0. The other taxa characterizing the initial stage WDC included *Pseudomonas*, *Stenotrophomonas*, *Cupriavidus*, and *Rhodobacteraceae*. Inoculant was seen as an increase in the relative abundance of *Bacillus* and changes in the relative abundances of other characterizing taxa, for example, *Brevundimonas* in the WDC + M treatment. The relative abundance of *Alcanivorax* was high in all treatments at days 60 and 120 and that of *Halomonas* relatively stable across all treatments over the whole experiment.

The 18S rRNA gene amplicon resulted in 1,820,720 reads that were classified into 458 OTUs. At day 120, Chao1 richness was higher in the not-inoculated treatments than in the WDC + M + P treatment, and Shannon diversity and Pielou’s evenness were highest (*P* ≤ 0.05) (Supplementary Table S3), indicating that over time, the inoculated fungal and bacterial consortium had affected the succession of the fungal community. At phylum level, the relative abundances of OTUs assigned to Ascomycota were high in all treatments, the relative abundances of Basidiomycota OTUs increased with time (Supplementary Figure S8), and communities at day 120 were distinct from those at days 0 and 60 (Figure 5B and Supplementary Figure S8). Based on the most abundant taxa, the community in the initial WDC was distinct from the rest, and communities at day 120 clustered together (Figure 6B).

Fungal taxa characteristic for different treatment–time combinations were identified in WDC and WDC + M at each time point and in WDC + P at day 120 (Supplementary Figure S8 and Supplementary Table S5). Unidentified *Nectriaceae* was most abundant in WDC at day 0 (Figure 6B). The relative abundances of *Acremonium* and unidentified *Nectriaceae* in WDC and those of *Phaeoacremonium* in WDC + M were high all through the experiment (Supplementary Table S5). Compared to WDC, inoculation approximately doubled the relative abundance of *Stachybotrys* and changed the relative abundances of other taxa, for example, *Microascus* in the WDC + M treatment at day 0.

Spearman correlations between abundant taxa and TOC removal rate were calculated to estimate the roles of the taxa in the bioremediation process. *Bacillus* and *Stachybotrys* that were in the inoculant correlated with TOC removal rate negatively and positively, respectively (*P* ≤ 0.05) (Table 1). In addition, four bacterial and two fungal taxa correlated positively and one fungus taxon negatively with TOC removal rate.

**TABLE 1:** Spearman correlation of total organic carbon removal rate and dominant taxa in remediating waste drill cuttings (WDCs).

Taxa	Spearman’s
*Bacillus*	−0.94**
*Brevundimonas*	0.82*
*Devosia*	0.87**
*Pseudochrobactrum*	0.74*
*Simiduia*	0.86**
*Phaeoacremonium*	0.80*
*Scedosporium*	0.92**
*Stachybotrys*	0.92**
*Cordycipitaceae*-unidentified	−0.73*

*P < 0.05; **P < 0.01. Only statistically significant correlations are shown.

### Partial Least Squares Path Modeling Analysis

**FIGURE 7:**
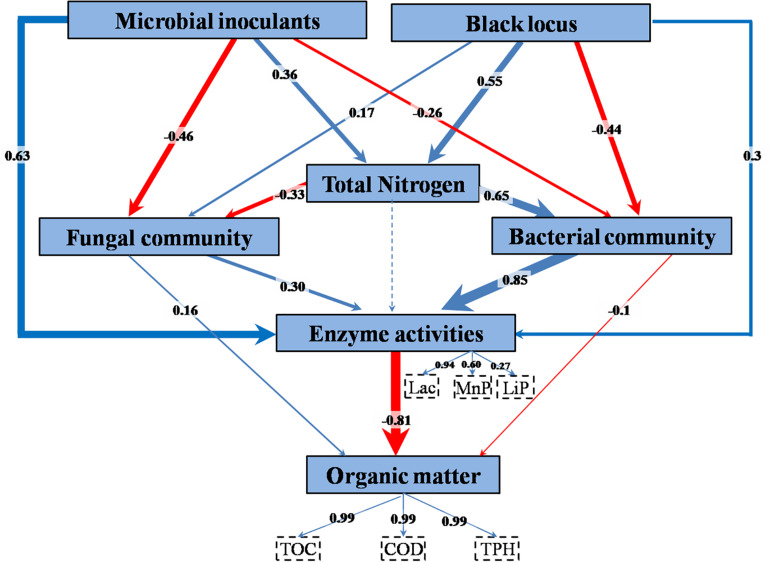
Partial least squares path model of remediating waste drill cuttings. A box represents an observed variable or a latent variable (i.e., a construct). The loadings for enzyme activities and organic removal rates that create the latent variables are shown in the dashed rectangles. Path coefficients are computed after 1,000 bootstraps and embodied in the width of the arrow, with blue and red indicating positive and negative effects, respectively. Dashed arrow shows a coefficient that did not differ significantly from 0 (*P* > 0.05). TN, total nitrogen content; Lac, laccase; MnP, manganese peroxidase; LiP, lignin peroxidase; TOC, total organic carbon content; COD, chemical oxygen demand; TPH, total petroleum hydrocarbon content.

The relationships among treatment factors and measured variables were studied using PLS-PM. TPH, TOC, and COD, the decreases of which were employed as indicators of bioremediation, were combined into organic matter content in the model (Figure 7 and Supplementary Table S6). The combined three enzyme activities had a significant direct effect on the organic matter content. The enzyme activities were directly affected by bacterial community, microbial inoculant, fungal community, and black locust. TN had an indirect effect on enzyme activities through its direct effects on the bacterial and fungal communities. The microbial inoculant had negative direct effects on the bacterial and fungal communities. The black locust had a negative direct effect on the bacterial community but a positive direct effect on the fungal community. Both the microbial inoculant and black locust had a positive direct effect on the TN.

## Discussion

We studied the efficiency of bioaugmentation and phytoremediation to remediate WDCs in an experiment where WDCs were treated with a fungal and bacterial consortium as microbial inoculant and *R. pseudoacacia* (black locust), either individually or in a combination. TPH and TOC contents in the WDC and COD of WDC extract were measured as indicators of the bioremediation process.

The synergistic combinations of bioaugmentation and phytoremediation may promote contaminant removal (Escalante-Espinosa et al., 2005; Huang et al., 2005; Lin et al., 2008; Dashti et al., 2009; Gurska et al., 2009). In our study, the TPH and TOC contents and COD decreased slightly over time in the control treatment, suggesting that indigenous microorganisms in WDCs had little pollutant degradation capability, yet some of the decreases may have been due to sorption of oil and chemicals to the PVC pots employed in the experiment. The TPH content and COD decreased most in the inoculated treatments, and TOC removal rate was lowest in the control, suggesting that inoculation with the bacterial and fungal consortium and the black locust growth had enhanced the degradation of pollutants in WDC. Inoculant increased the contaminant removal more than the black locust, yet the inoculant and black locust combination was the most efficient remediation treatment possibly due to the root exudates in rhizosphere, which are known to stimulate contaminant-degrading microorganisms and to enhance biodegradation ability (Grayston et al., 1997; Walker et al., 2003; Kechavarzi et al., 2007; Neumann, 2007). The plant root system is not expected to spread in the soil as quickly as the microbial inoculum, which may explain why phytoremediation alone would need a longer treatment time than bioaugmentation, especially when the plant growth is affected by pollutants (Zhang et al., 2020).

In heavily hydrocarbon-contaminated environments, for example, in petroleum-contaminated soil, the C/N ratio imbalance due to high carbon and low nitrogen contents can affect microbial activity and bioremediation efficiency. Hence, the amount of N supplied is of vital importance for bioremediation (Rojas-Avelizapa et al., 2007; Alavi et al., 2014; Taiwo et al., 2015). In agreement, the N content and TOC removal rate correlated positively in our study, indicating that the lower C/N ratio had increased the efficiency of remediation. Direct fertilization could rapidly increase the amount of N in soil, and in heavily contaminated environments, the need for swift pollutant removal is paramount; biostimulation has been used successfully, for example, in remediating Exxon Valdez oil spill (Lindstrom et al., 1991; Venosa et al., 2010). However, it may bring about additional environmental problems for eutrophication of water bodies. In contrast, taking advantage of biological nitrogen fixation (BNF) is efficient and inexpensive and a sustainable way to increase the amount of N in soil. In our study, the black locust, a legume tree that forms symbiotically nitrogen-fixing nodules with rhizobia bacteria (Gilman and Watson, 1994; Bolat et al., 2015), increased soil N content in the black locust alone and black locust in combination with the inoculant treatments. The abundance of free-living nitrogen-fixing microorganisms was lower in black locust alone than in black locust in combination with the inoculant. In bioremediation, N content increase can be due to the activity of free-living nitrogen-fixing bacteria (Al-Mailem et al., 2019). In our study, possibly the inoculation had stimulated BNF by free-living microorganisms. The relative abundances of *Halomonas* and *Bacillus* that were in the inoculum, and those of *Pseudomonas* and *Lysobacter*, all of which include free-living nitrogen fixers (Seldin et al., 1983; Llamas et al., 2006; Yan et al., 2008), were higher with than without inoculum or plant, suggesting that these taxa may have influenced N content during the remediation process.

The lignin-degrading enzymes, including Lac, MnP, and LiP, play an important role in the bioremediation of pesticides, polycyclic aromatic hydrocarbons, and other xenobiotics (Karigar and Rao, 2011). Lac is abundant in soil and functions outside of the cells to facilitate bacterial and fungal degradation of pesticides, Polycyclic aromatic hydrocarbons (PAHs), and lignin (Yanto et al., 2017; Dandare et al., 2019; Muhammad et al., 2019). In our study, Lac activity correlated positively with the TOC removal rate and was highest in the inoculated treatments, suggesting that the activity was either brought on or stimulated by the inoculated bacterial and fungal consortium.

Both the bacterial and fungal richness and bacterial diversity and evenness indices increased in all the treatments over time, whereas the fungal diversity and evenness increased only in the not-inoculated treatments. As the contaminant removal was highest in the inoculated treatments, the less even fungal communities in the inoculated treatments might have been due to increased growth of contaminant-degrading fungi or contaminant-sensitive fungi. The dominant taxa that characterized the differences between bioremediation treatments and control included bacterial genera *Pseudochrobactrum*, *Brevundimonas*, and *Bacillus* and fungal genera *Scedosporium*, *Stachybotrys*, *Microascus*, and *Acremonium*. *Pseudochrobactrum* can degrade phenols (Mao et al., 2015), halogenated aromatics (Liu et al., 2016), and lignin (Shannon et al., 2017), and *Brevundimonas* has the ability to degrade phenols (Zhang et al., 2018). The dominant fungal genera have the ability to degrade lignin, cellulose, and PAHs (Santos et al., 2006; López-González et al., 2015). In our study, *Brevundimonas*, *Phaeoacremonium*, *Scedosporium*, and *Stachybotrys*, members of the inoculated consortium, correlated positively with TOC removal rate, suggesting that these four genera played major roles in WDC remediation. Out of the other detected inoculant consortium members, *Bacillus* peaked at day 0 and correlated negatively with TOC removal, suggesting that the adaptive ability of the inoculated *Bacillus* strain was poor in WDC. However, since relative abundances are not informative of absolute abundances (McLaren et al., 2019), determining the contribution of the inoculated strains would require quantitative analyses, for example, qPCR. Due to the differences in degradation efficiency between strains, determining the absolute contribution of the strains seems unattainable in an *in vivo* experiment.

The results above suggested that the inoculated consortium and the planting of black locust increased N content, enzyme activities, and macromolecular organic pollutant (SPF and SL) removal rate and affected microbial community. In a contaminated environment, the major factors in biological degradation of organic pollutants are the intracellular and extracellular enzymes produced by microbes (Tünde and Tien, 2000; Karigar and Rao, 2011). Phytoremediation, biostimulation, and bioaugmentation commonly increased the efficiency of bioremediation by increasing the activities of degradation enzymes (Mauricio-Gutiérrez et al., 2014; Chandanshive et al., 2018; Song et al., 2019). In agreement, in our study, the PLS-PM analysis showed that among the complex interrelationships between the factors, enzyme activities, especially that of Lac, played a key role in the bioremediation process. The enzyme activities were mostly governed by inoculated consortium and bacterial community, and black locust affected the bioremediation mainly through its effect on N content that further affected bacterial and fungal communities.

## Conclusion

Compared with natural attenuation in the control treatment, bioaugmentation and phytoremediation individually and especially in combination enhanced contaminant removal from WDCs. The microbial inoculant affected the soil fungal and bacterial communities directly and planting of black locust indirectly *via* soil N content, yet most of their effect on the bioremediation process was indirect through enzyme activities.

## Data Availability Statement

The sequences have been submitted to the NCBI Sequence Read Archive under accession numbers PRJNA601856 and PRJNA609003.

## Author Contributions

HL, QC, and LD designed the experiments. LC and MH collected samples. HL, YL, and LZ carried out the experiments. KZ and XY analyzed the experimental results. YG analyzed the sequencing data and developed analysis tools. XZ assisted with Illumina sequencing. HL, EW, QC, and PP wrote the manuscript. All authors contributed to the article and approved the submitted version.

## Conflict of Interest

HL, LC, and MH were employed by the company Safety and Environmental Protection Quality Supervision and Testing Research Institute, CNPC Chuanqing Drilling Engineering Co. Ltd. The remaining authors declare that the research was conducted in the absence of any commercial or financial relationships that could be construed as a potential conflict of interest.

## Supplementary Material

Supplementary Figure 1 Total organic carbon (TOC) contents of soil extracts from waste drill cuttings (WDCs). WDCs were incubated in a greenhouse for 120 days with and without black locust (*Robinia pseudoacacia*) plant and with or without bacterial and fungal consortium inoculant. Statistically significant differences are indicated with asterisks: ^∗^*P* < 0.05; ^∗∗^*P* < 0.01; ^∗∗∗^*P* < 0.001; ^****^*P* < 0.0001. The boxes show mean, lower and upper hinges indicate the first and third quartiles, and the whiskers indicate the ranges 1.5 times the interquartile range.

Supplementary Figure 2 Chemical oxygen demand (COD) of soil extracts from waste drill cuttings (WDCs). WDCs were incubated in a greenhouse for 120 days with and without black locust (*Robinia pseudoacacia*) plant and with or without bacterial and fungal consortium inoculant. Statistically significant differences are indicated with asterisks: ^∗^*P* < 0.05; ^∗∗^*P* < 0.01; ^∗∗∗^*P* < 0.001; ^****^*P* < 0.0001. The boxes show mean, lower and upper hinges indicate the first and third quartiles, and the whiskers indicate the ranges 1.5 times the interquartile range.

Supplementary Figure 3 Chemical oxygen demand (COD) of soil extracts from waste drill cuttings (WDCs) versus that in natural soil. WDCs were incubated in a greenhouse for 120 days with and without black locust (*Robinia pseudoacacia*) plant and with or without bacterial and fungal consortium inoculant. WDC, no plant and no inoculant; WDC + M, no plant and inoculant; WDC + P, plant and no inoculant; WDC + P + M, plant and inoculant. Statistically significant differences are indicated with asterisks: ^∗^*P* < 0.05; ^∗∗^*P* < 0.01; ^∗∗∗^*P* < 0.001; ^****^*P* < 0.0001. The boxes show mean, lower and upper hinges indicate the first and third quartiles, and the whiskers indicate the ranges 1.5 times the interquartile range.

Supplementary Figure 4 The germination rate of red clover seeds in waste drill cuttings (WDCs) versus that in natural soil. WDCs were incubated in a greenhouse for 120 days with and without black locust (*Robinia pseudoacacia*) plant and with or without bacterial and fungal consortium inoculant. WDC, no plant and no inoculant; WDC + M, no plant and inoculant; WDC + P, plant and no inoculant; WDC + P + M, plant and inoculant. Statistically significant differences are indicated with asterisks: ^∗^*P* < 0.05; ^∗∗^*P* < 0.01; ^∗∗∗^*P* < 0.001; ^****^*P* < 0.0001. The boxes show mean, lower and upper hinges indicate the first and third quartiles, and the whiskers indicate the ranges 1.5 times the interquartile range.

Supplementary Figure 5 Activity of manganese peroxidase (MnP) in waste drill cuttings (WDCs). WDCs were incubated in a greenhouse for 120 days with and without black locust (*Robinia pseudoacacia*) plant and with or without bacterial and fungal consortium inoculant. Statistically significant differences are indicated with asterisks: ^∗^*P* < 0.05; ^∗∗^*P* < 0.01; ^∗∗∗^*P* < 0.001; ^****^*P* < 0.0001. The boxes show mean, lower and upper hinges indicate the first and third quartiles, and the whiskers indicate the ranges 1.5 times the interquartile range.

Supplementary Figure 6 Activity of lignin peroxidase (LiP) in waste drill cuttings (WDCs). WDCs were incubated in a greenhouse for 120 days with and without black locust (*Robinia pseudoacacia*) plant and with or without bacterial and fungal consortium inoculant. Statistically significant differences are indicated with asterisks: ^∗^*P* < 0.05; ^∗∗^*P* < 0.01; ^∗∗∗^*P* < 0.001; ^****^*P* < 0.0001. The boxes show mean, lower and upper hinges indicate the first and third quartiles, and the whiskers indicate the ranges 1.5 times the interquartile range.

Supplementary Figure 7 Composition of bacterial community in waste drill cuttings (WDCs). WDCs were incubated in a greenhouse for 120 days with and without black locust (*Robinia pseudoacacia*) plant and with or without bacterial and fungal consortium inoculant. The relative abundances at phylum level on the left. Taxa characterizing the differences between treatments on the right, identified using the linear discriminant analysis (LDA) effect size (LEfSe) method. WDC, no plant and no inoculant; WDC + M, no plant and inoculant; WDC + P, plant and no inoculant; WDC + M + P, plant and inoculant.

Supplementary Figure 8 Composition of fungal community in waste drill cuttings (WDCs). WDCs were incubated in a greenhouse for 120 days with and without black locust (*Robinia pseudoacacia*) plant and with or without bacterial and fungal consortium inoculant. The relative abundances at phylum level on the left. Taxa characterizing the differences between treatments on the right, identified using the linear discriminant analysis (LDA) effect size (LEfSe) method. WDC, no plant and no inoculant; WDC + M, no plant and inoculant; WDC + P, plant and no inoculant; WDC + M + P, plant and inoculant.

Supplementary Table 1 Physicochemical properties of natural soil (NS) and waste drill cuttings prior the experiment [waste drill cutting (WDC)].

Supplementary Table 2 Statistical significances of the differences between inoculation and plant treatments over time and their interactions in three-way mixed ANOVA. ^∗^ indicates *P* < 0.05.

Supplementary Table 3 Richness and diversity of the bacterial and fungal communities in remediating waste drill cuttings (WDCs). WDCs were incubated in a greenhouse for 120 days with and without black locust (*Robinia pseudoacacia*) plant and with or without bacterial and fungal consortium inoculant.

Supplementary Table 4 Relative abundances of dominant bacterial taxa in waste drill cuttings (WDCs). WDCs were incubated in a greenhouse for 120 days with and without black locust (*Robinia pseudoacacia*) plant and with or without bacterial and fungal consortium inoculant.

Supplementary Table 5 Relative abundance of dominant fungal taxa in waste drill cuttings (WDCs). WDCs were incubated in a greenhouse for 120 days with and without black locust (*Robinia pseudoacacia*) plant and with or without bacterial and fungal consortium inoculant.

Supplementary Table 6 The direct and indirect relationships between variables in the partial least squares path model (PLS-PM) of remediating waste drill cuttings. The path coefficients are calculated by PLS-PM after 1,000 bootstraps.
