# Supplementary material for: Combined Microbial Consortium Inoculation and Black Locust Planting Is Effective in the Bioremediation of Waste Drill Cuttings
Source: Front Microbiol. 2020 Sep 30;11:536787. doi: 10.3389/fmicb.2020.536787 (PMC7555700; doi:10.3389/fmicb.2020.536787)
Supplement: Supplementary Figure 1 — Total organic carbon (TOC) contents of soil extracts from waste drill cuttings (WDCs). WDCs were incubated in a greenhouse for 120 days with and without black locust (Robinia pseudoacacia) plant and with or without bacterial and fungal consortium inoculant. Statistically significant differences are indicated with asterisks: ∗P < 0.05; ∗∗P < 0.01; ∗∗∗P < 0.001; ****P < 0.0001. The boxes show mean, lower and upper hinges indicate the first and third quartiles, and the whiskers indicate the ranges 1.5 times the interquartile range. [file Data_Sheet_1.doc]

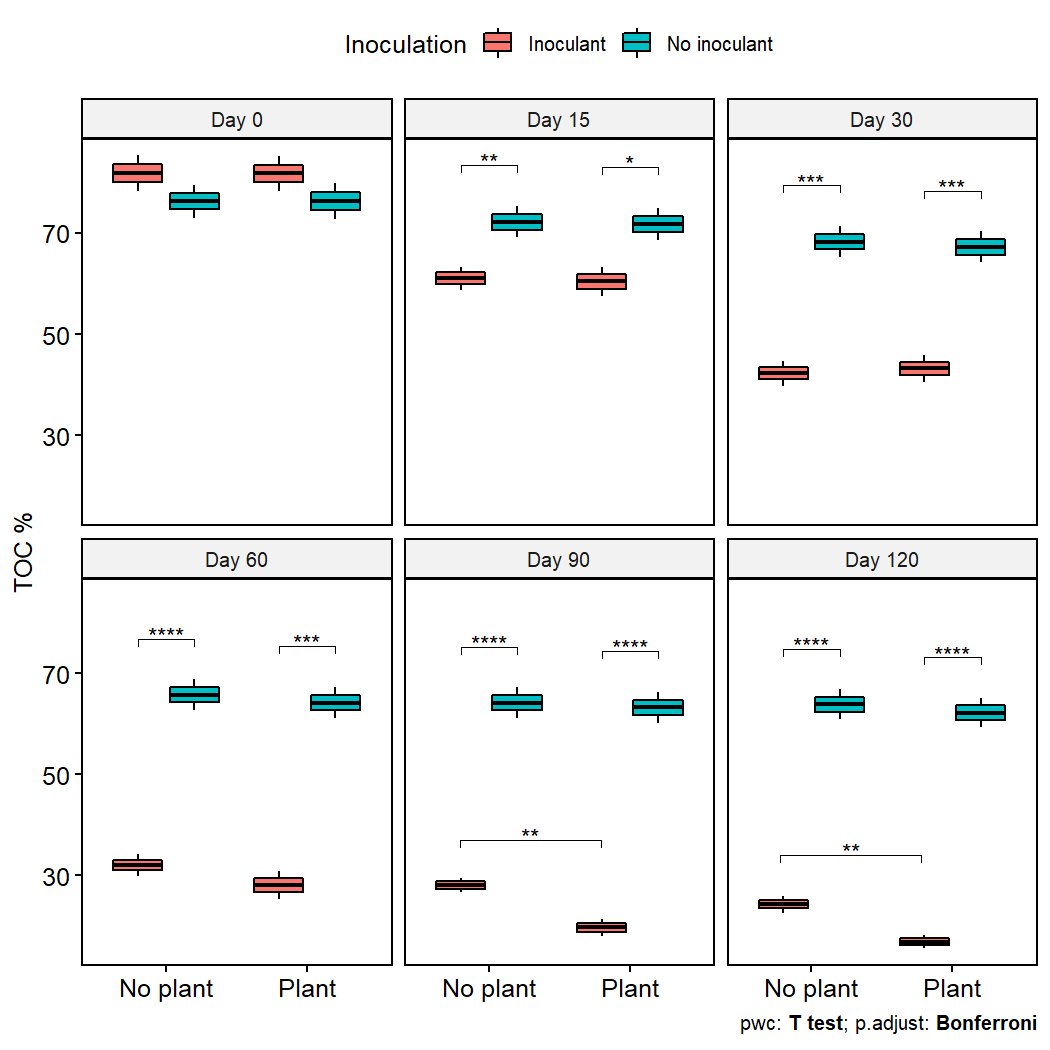


Fig. S1 Total organic carbon (TOC) contents of soil extracts from waste drill cuttings (WDC). WDC was incubated in a greenhouse for 120 days with and without black locust (*Robinia pseudoacacia*) plant and with or without bacterial and fungal consortium inoculant. Statistically significant differences are indicated with asterisks: *, *P* < 0.05; **, *P* < 0.01; ***, *P* < 0.001; ****, *P* < 0.0001. The boxes show mean, lower and upper hinges indicate the first and third quartiles, and the whiskers indicate the ranges 1.5 times the inter-quartile range.


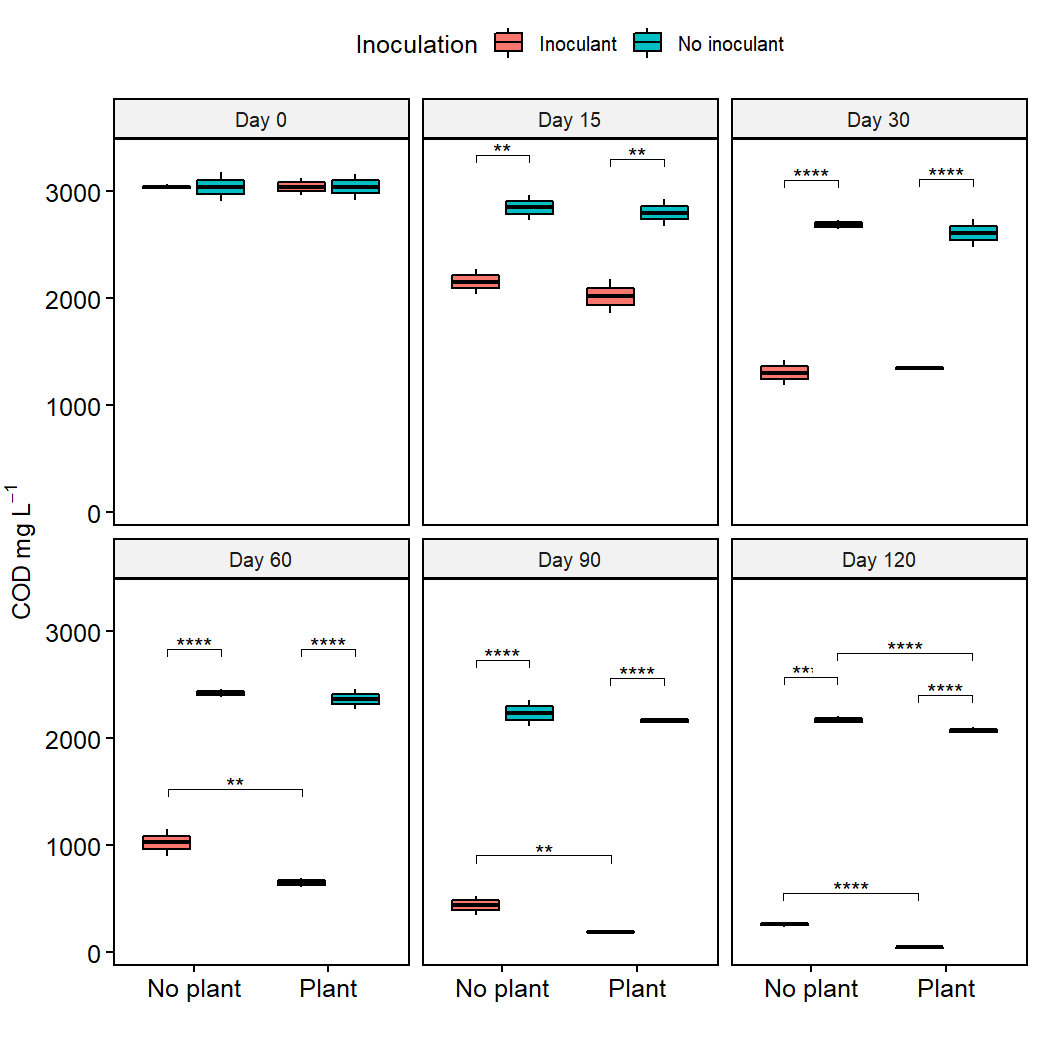


Fig. S2 Chemical oxygen demand (COD) of soil extracts from waste drill cuttings (WDC). WDC was incubated in a greenhouse for 120 days with and without black locust (*Robinia pseudoacacia*) plant and with or without bacterial and fungal consortium inoculant. Statistically significant differences are indicated with asterisks: **, *P* < 0.01; ****, *P* < 0.0001. The boxes show mean, lower and upper hinges indicate the first and third quartiles, and the whiskers indicate the ranges 1.5 times the inter-quartile range.


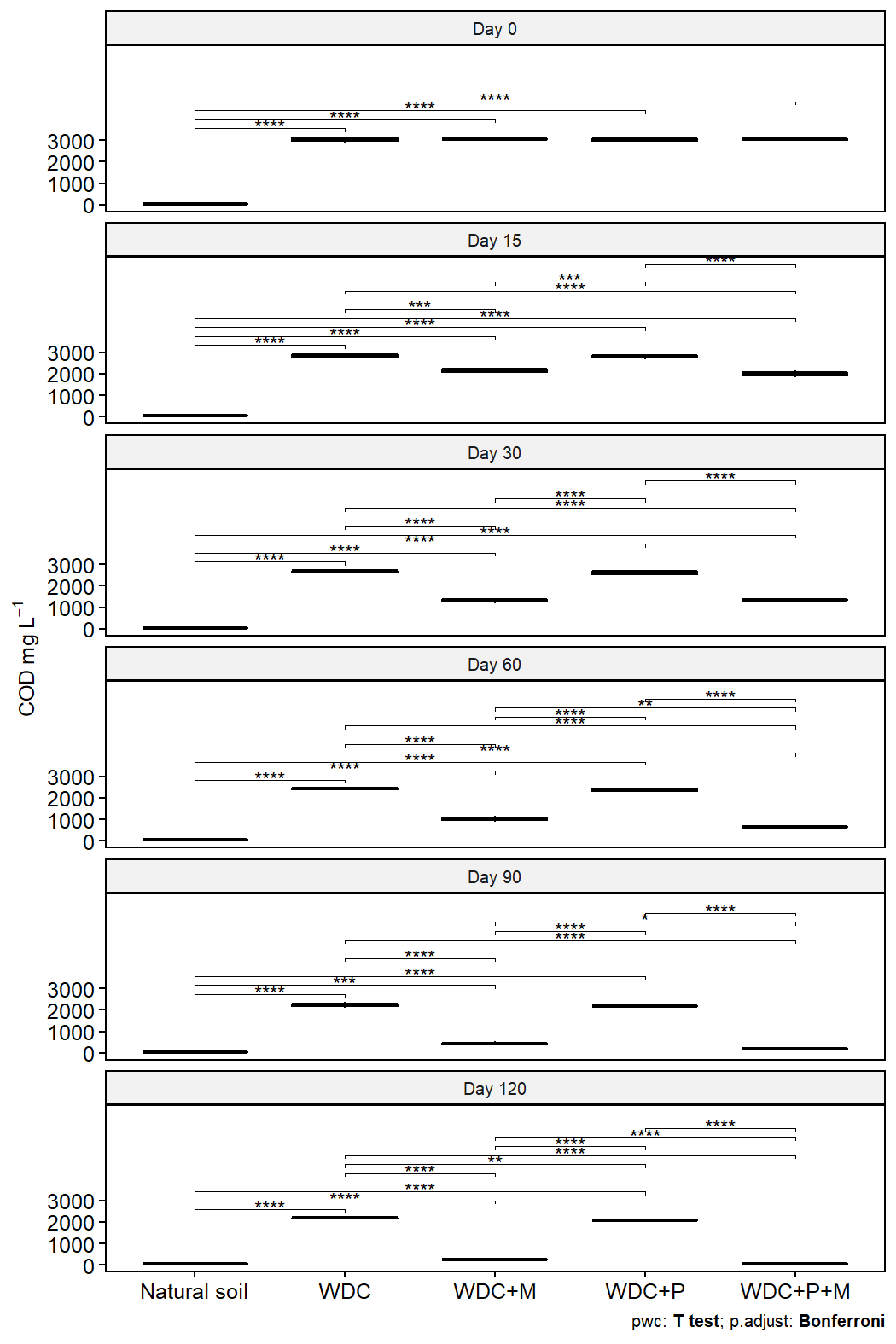


Fig. S3 Chemical oxygen demand (COD) of soil extracts from waste drill cuttings (WDC) versus that in natural soil. WDC was incubated in a greenhouse for 120 days with and without black locust (*Robinia pseudoacacia*) plant and with or without bacterial and fungal consortium inoculant. WDC, no plant and no inoculant; WDC+M, no plant and inoculant; WDC+P, plant and no inoculant; WDC+P+M, plant and inoculant. Statistically significant differences are indicated with asterisks: *, *P* < 0.05; **, *P* < 0.01; ***, *P* < 0.001; ****, *P* < 0.0001. The boxes show mean, lower and upper hinges indicate the first and third quartiles, and the whiskers indicate the ranges 1.5 times the inter-quartile range.


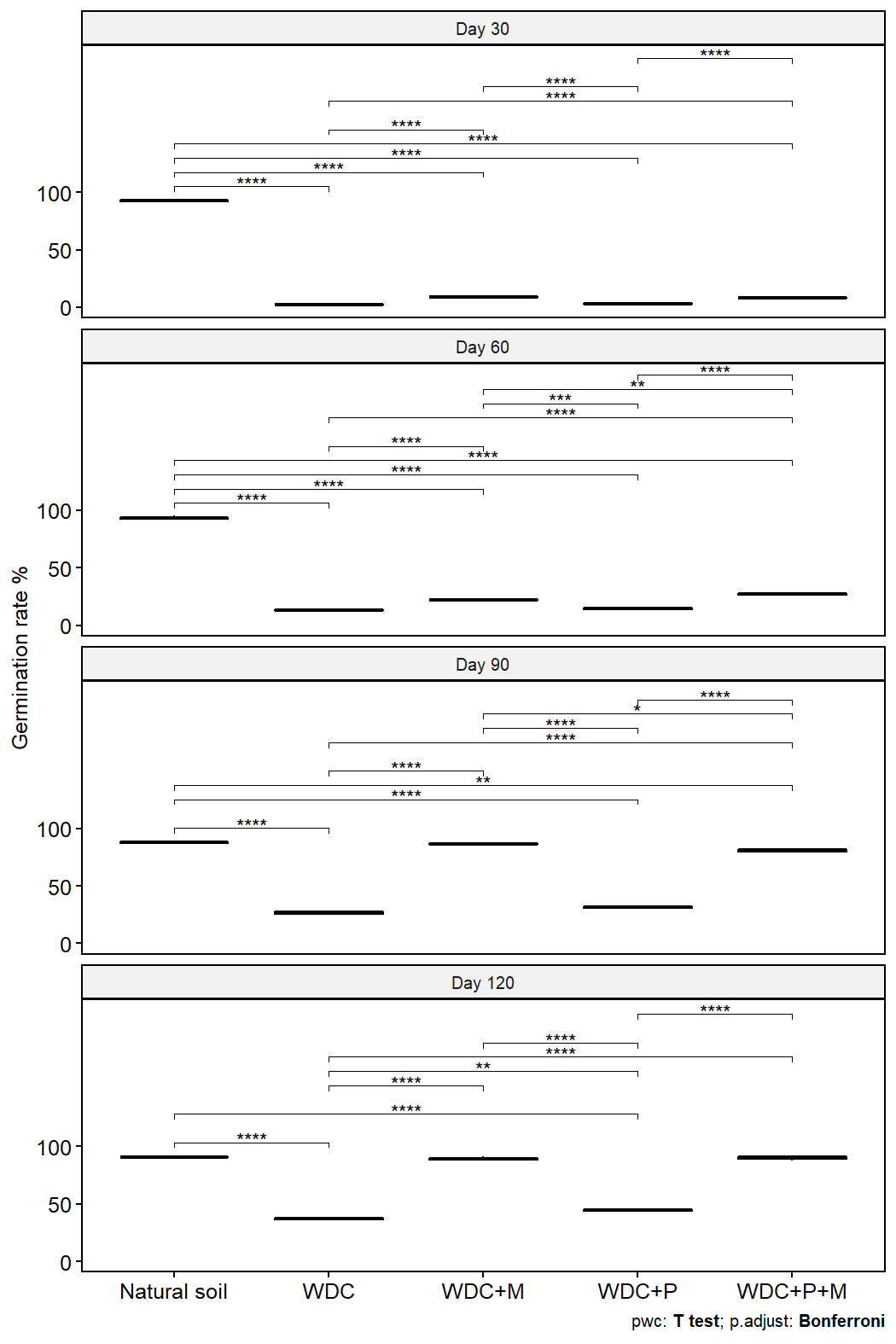


Fig. S4 The germination rate of red clover seeds in waste drill cuttings (WDC) versus that in natural soil. WDC was incubated in a greenhouse for 120 days with and without black locust (*Robinia pseudoacacia*) plant and with or without bacterial and fungal consortium inoculant. WDC, no plant and no inoculant; WDC+M, no plant and inoculant; WDC+P, plant and no inoculant; WDC+P+M, plant and inoculant. Statistically significant differences are indicated with asterisks: *, *P* < 0.05; **, *P* < 0.01; ***, *P* < 0.001; ****, *P* < 0.0001. The boxes show mean, lower and upper hinges indicate the first and third quartiles, and the whiskers indicate the ranges 1.5 times the inter-quartile range.


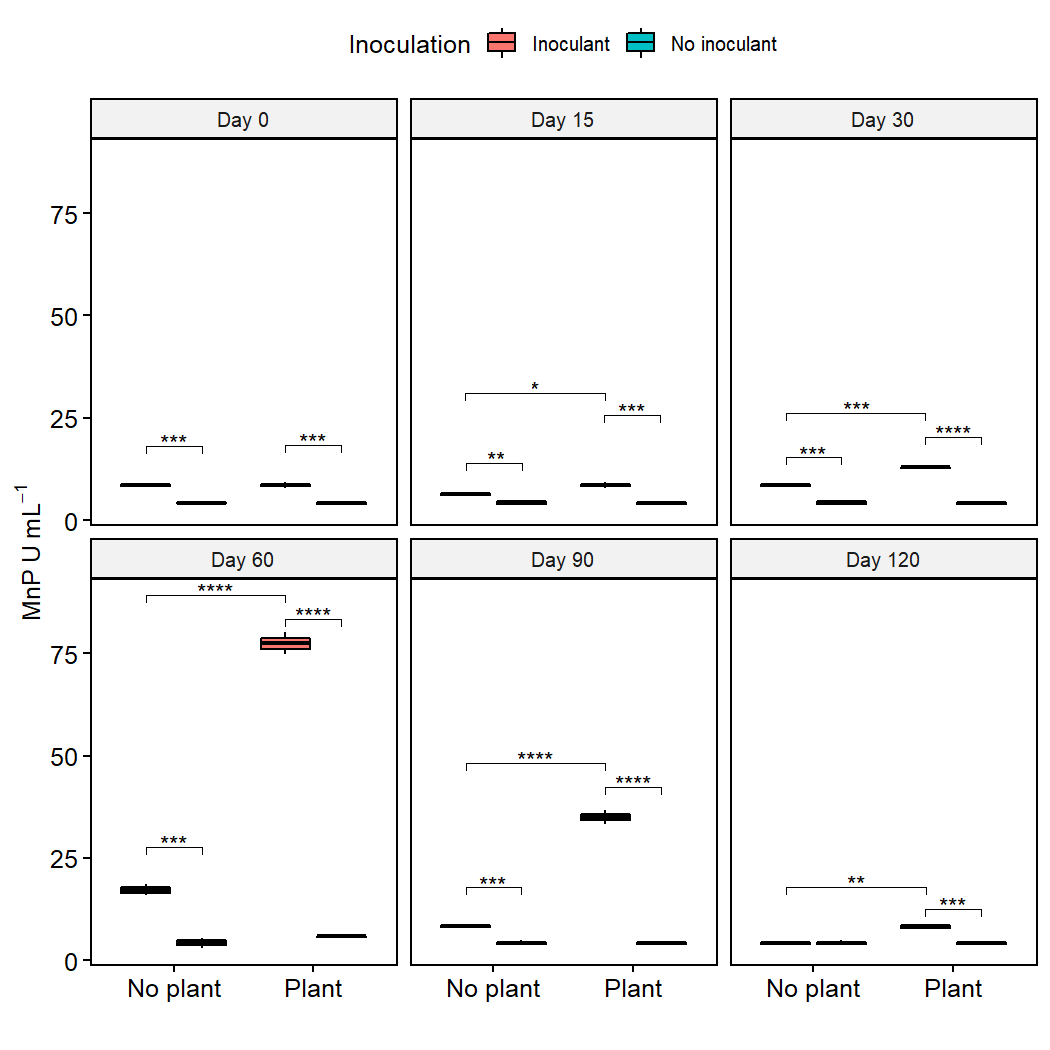


Fig. S5 Activity of manganese peroxidase (MnP) in waste drill cuttings (WDC). WDC was incubated in a greenhouse for 120 days with and without black locust (*Robinia pseudoacacia*) plant and with or without bacterial and fungal consortium inoculant. Statistically significant differences are indicated with asterisks: *, *P* < 0.05; **, *P* < 0.01; ***, *P* < 0.001; ****, *P* < 0.0001. The boxes show mean, lower and upper hinges indicate the first and third quartiles, and the whiskers indicate the ranges 1.5 times the inter-quartile range.


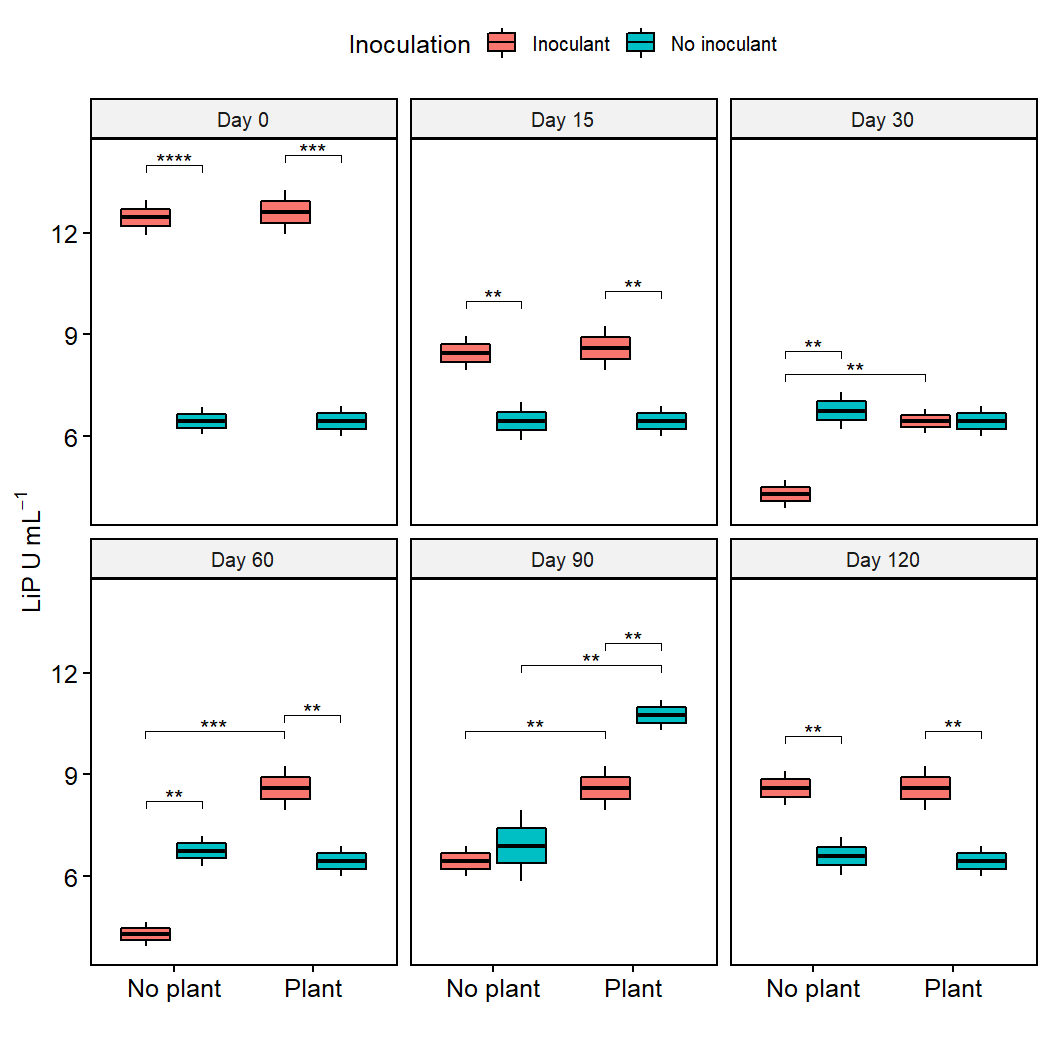


Fig. S6 Activity of lignin peroxidase (LiP) in waste drill cuttings (WDC). WDC was incubated in a greenhouse for 120 days with and without black locust (*Robinia pseudoacacia*) plant and with or without bacterial and fungal consortium inoculant. Statistically significant differences are indicated with asterisks: **, *P* < 0.01; ***, *P* < 0.001; ****, *P* < 0.0001. The boxes show mean, lower and upper hinges indicate the first and third quartiles, and the whiskers indicate the ranges 1.5 times the inter-quartile range.


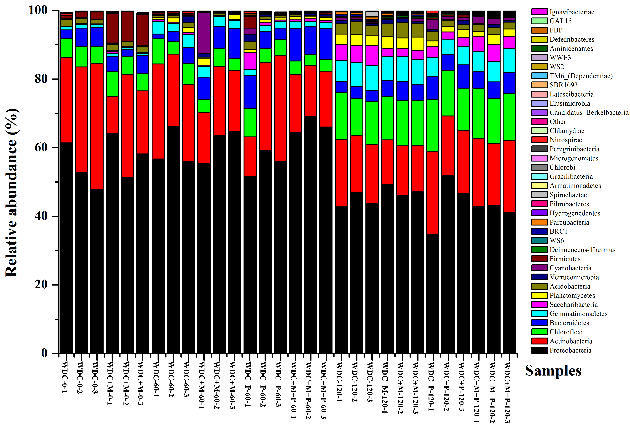

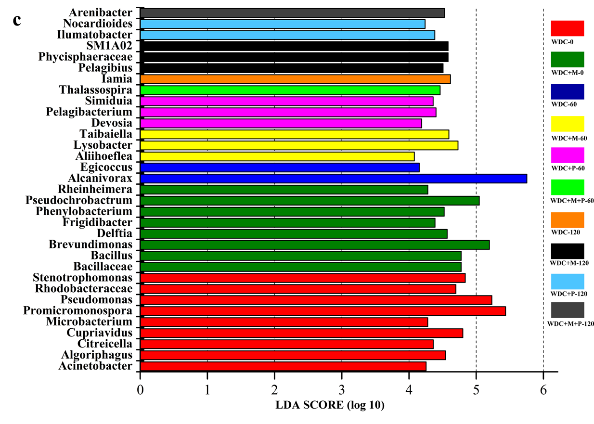


Fig.S7 Composition of bacterial community in waste drill cuttings (WDC). WDC was incubated in a greenhouse for 120 days with and without black locust (*Robinia pseudoacacia*) plant and with or without bacterial and fungal consortium inoculant. The relative abundances at phylum level on the left. Taxa characterizing the differences between treatments on the right, identified using the linear discriminant analysis (LDA) effect size (LEfSe) method. WDC, no plant and no inoculant; WDC+M, no plant and inoculant; WDC+P, plant and no inoculant; WDC+M+P, plant and inoculant.


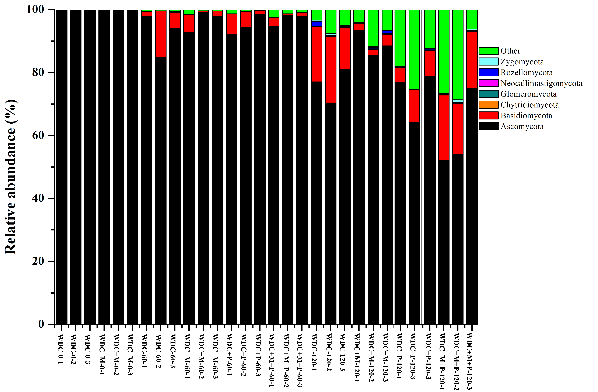

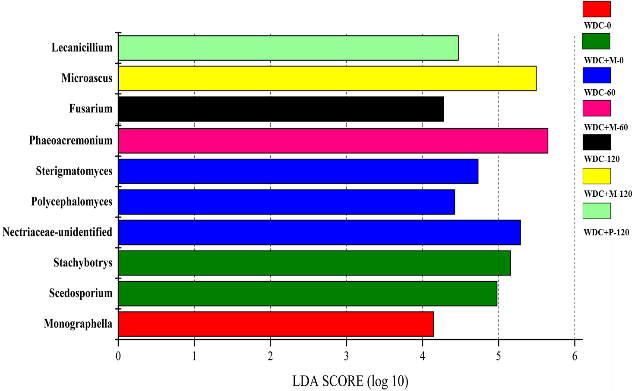


Fig. S8 Composition of fungal community in waste drill cuttings (WDC). WDC was incubated in a greenhouse for 120 days with and without black locust (*Robinia pseudoacacia*) plant and with or without bacterial and fungal consortium inoculant. The relative abundances at phylum level on the left. Taxa characterizing the differences between treatments on the right, identified using the linear discriminant analysis (LDA) effect size (LEfSe) method. WDC, no plant and no inoculant; WDC+M, no plant and inoculant; WDC+P, plant and no inoculant; WDC+M+P, plant and inoculant.

Table S1. Physicochemical properties of natural soil (NS) and waste drill cuttings prior the experiment (WDC).

| Property |  | NS | WDC |
| --- | --- | --- | --- |
| Water content (%) | | 25 | 35 |
| Density（g cm^-3^） | | ND | 1.82 |
| pH | | 7.2 | 8.05 |
| TPH（g kg^-1^） | | ND | 6.11 |
| TOC（%） | | ND | 2.63 |
| Total N（mg kg^-1^） | | ND | 301.23 |
| Total P（mg kg^-1^） | | ND | 233.34 |
| C/N ratio | | ND | 87 |
| COD of extract（mg L^-1^） | | 40.6 | 3042.68 |
| Clover seed germination (%) | | 89.33 | 0 |

ND: not determined

Table S2. Statistical significances of the differences between inoculation and plant treatments over time and their interactions in three-way mixed ANOVA. * indicates p<0.05.

| Variable | Effect | DFn | DFd | F | p |
| --- | --- | --- | --- | --- | --- |
| TPH | Inoculation | 1 | 8 | 197.741 | * |
|  | Plant | 1 | 8 | 1.22 |  |
|  | time | 5 | 40 | 2251.156 | * |
|  | Inoculation:Plant | 1 | 8 | 0.294 |  |
|  | Inoculation:time | 5 | 40 | 587.045 | * |
|  | Plant:time | 5 | 40 | 5.276 | * |
|  | Inoculation:Plant:time | 5 | 40 | 3.12 | * |
| TOC | Inoculation | 1 | 8 | 241.426 | * |
|  | Plant | 1 | 8 | 1.74 |  |
|  | time | 5 | 40 | 7908.669 | * |
|  | Inoculation:Plant | 1 | 8 | 0.532 |  |
|  | Inoculation:time | 5 | 40 | 3244.709 | * |
|  | Plant:time | 5 | 40 | 44.543 | * |
|  | Inoculation:Plant:time | 5 | 40 | 31.204 | * |
| COD | Inoculation | 1 | 8 | 798.681 | * |
|  | Plant | 1 | 8 | 5.903 | * |
|  | time | 5 | 40 | 2405.801 | * |
|  | Inoculation:Plant | 1 | 8 | 1.196 |  |
|  | Inoculation:time | 5 | 40 | 638.355 | * |
|  | Plant:time | 5 | 40 | 8.076 | * |
|  | Inoculation:Plant:time | 5 | 40 | 6.21 | * |
| TN | Inoculation | 1 | 8 | 180.742 | * |
|  | Plant | 1 | 8 | 356.863 | * |
|  | time | 5 | 40 | 12725.27 | * |
|  | Inoculation:Plant | 1 | 8 | 6.953 | * |
|  | Inoculation:time | 5 | 40 | 1177.988 | * |
|  | Plant:time | 5 | 40 | 19093.46 | * |
|  | Inoculation:Plant:time | 5 | 40 | 1149.173 | * |
| MnP | Inoculation | 1 | 8 | 860.507 | * |
|  | Plant | 1 | 8 | 368.211 | * |
|  | time | 5 | 40 | 3134.018 | * |
|  | Inoculation:Plant | 1 | 8 | 346.063 | * |
|  | Inoculation:time | 5 | 40 | 2913.578 | * |
|  | Plant:time | 5 | 40 | 1767.177 | * |
|  | Inoculation:Plant:time | 5 | 40 | 1607.086 | * |
| LiP | Inoculation | 1 | 8 | 16.948 | * |
|  | Plant | 1 | 8 | 10.704 | * |
|  | time | 5 | 40 | 993.197 | * |
|  | Inoculation:Plant | 1 | 8 | 2.5 |  |
|  | Inoculation:time | 5 | 40 | 1275.01 | * |
|  | Plant:time | 5 | 40 | 255.866 | * |
|  | Inoculation:Plant:time | 5 | 40 | 199.178 | * |
| Lac | Inoculation | 1 | 8 | 584.069 | * |
|  | Plant | 1 | 8 | 37.326 | * |
|  | time | 5 | 40 | 4001.446 | * |
|  | Inoculation:Plant | 1 | 8 | 58.871 | * |
|  | Inoculation:time | 5 | 40 | 756.039 | * |
|  | Plant:time | 5 | 40 | 46.754 | * |
|  | Inoculation:Plant:time | 5 | 40 | 61.67 | * |
| Germination | Inoculation | 1 | 8 | 1876.358 | * |
|  | Plant | 1 | 8 | 6.42 | * |
|  | time | 4 | 32 | 14579.01 | * |
|  | Inoculation:Plant | 1 | 8 | 7.715 | * |
|  | Inoculation:time | 4 | 32 | 2706.581 | * |
|  | Plant:time | 4 | 32 | 18.349 | * |
|  | Inoculation:Plant:time | 4 | 32 | 33.613 | * |

Table S3. Richness and diversity of the bacterial and fungal communities in remediating waste drill cuttings (WDC). WDC was incubated in a greenhouse for 120 days with and without black locust (*Robinia pseudoacacia*) plant and with or without bacterial and fungal consortium inoculant.

| Treatment | Bacteria | |  | Fungi | |  |
| --- | --- | --- | --- | --- | --- | --- |
|  | Chao1 | Shannon | Pielou’s | Chao1 | Shannon | Pielou’s |
| WDC (Day 0) | 1939.71 c | 7.90 e | 0.7268 c | 308.83 bc | 4.06 b | 0.4900 c |
| WDC+M (Day 0) | 1546.12 c | 7.16 f | 0.6783 d | 263.29 c | 3.52 b | 0.4343 cd |
| WDC (Day 60) | 2987.98 ab | 8.52 d | 0.7520 bc | 384.66 b | 4.17 b | 0.4828 cd |
| WDC+M (Day 60) | 3130.96 ab | 8.75 cd | 0.7661 bc | 347.35 bc | 3.56 b | 0.4220 d |
| WDC+P (Day 60) | 3416.19 a | 8.95 bc | 0.7794 b | 338.12 bc | 3.73 b | 0.4412 cd |
| WDC+M+P (Day 60) | 3092.68 ab | 8.83 ab | 0.7790 b | 355.81 bc | 4.10 b | 0.4742 cd |
| WDC (Day 120) | 2811.10 b | 9.59 a | 0.8312 a | 493.76 a | 5.53 a | 0.6166 a |
| WDC+M (Day 120) | 3138.63 ab | 9.69 a | 0.8337 a | 427.33 ab | 3.98 b | 0.4523 cd |
| WDC+P (Day 120) | 3004.16 ab | 9.75 a | 0.8379 a | 503.51 a | 5.15 a | 0.5716 b |
| WDC+M+P (Day 120) | 2671.34 b | 9.36 ab | 0.8110 ab | 389.41 b | 4.16 b | 0.4797 cd |

WDC, no plant and no inoculant; WDC+M, no plant and inoculant; WDC+P, plant and no inoculant; WDC+M+P, plant and inoculant. Different letters after the numbers in a column indicate statistically significant difference at *P <* 0.05.

Table S4 Relative abundances of dominant bacterial taxa in waste drill cuttings (WDC). WDC was incubated in a greenhouse for 120 days with and without black locust (*Robinia pseudoacacia*) plant and with or without bacterial and fungal consortium inoculant.

| Taxon | Relative abundance (%) | | | | | | | | | |
| --- | --- | --- | --- | --- | --- | --- | --- | --- | --- | --- |
|  | WDC  (Day 0) | WDC+M  (Day 0) | WDC  (Day 60) | WDC+M  (Day 60) | WDC+P  (Day 60) | WDC+M+P  (Day 60) | WDC  (Day 120) | WDC+M  (Day 120) | WDC+P  (Day 120) | WDC+M+P  (Day 120) |
| *Promicromonospora* | 18.51 | 10.56 | 11.73 | 8.07 | 7.20 | 11.97 | 4.42 | 2.74 | 5.52 | 3.82 |
| *Pseudomonas* | 12.05 | 6.10 | 0.66 | 1.25 | 0.69 | 1.85 | 0.23 | 0.28 | 0.85 | 7.97 |
| *Stenotrophomonas* | 4.93 | 0.71 | 0.01 | 0.11 | 0.01 | 0.07 | 0.02 | 0.00 | 0.04 | 0.18 |
| *Halomonas* | 4.71 | 3.14 | 3.85 | 4.89 | 5.17 | 4.21 | 2.97 | 5.35 | 2.62 | 2.96 |
| *Rhodobacteraceae* | 4.00 | 3.99 | 0.10 | 0.16 | 0.12 | 0.16 | 0.03 | 0.02 | 0.07 | 0.10 |
| *Algoriphagus* | 2.31 | 1.55 | 0.85 | 1.07 | 1.33 | 1.30 | 0.07 | 0.11 | 0.19 | 0.21 |
| *Alcanivorax* | 1.76 | 1.16 | 40.45 | 31.46 | 34.57 | 30.07 | 16.74 | 17.77 | 19.33 | 13.94 |
| *Microbacterium* | 1.31 | 0.96 | 0.41 | 0.55 | 0.68 | 0.60 | 0.22 | 0.35 | 0.39 | 0.73 |
| *Phenylobacterium* | 1.26 | 2.21 | 0.24 | 0.32 | 0.30 | 0.25 | 0.07 | 0.21 | 0.06 | 0.14 |
| *Brevundimonas* | 1.05 | 10.31 | 0.25 | 3.82 | 3.51 | 0.38 | 0.08 | 0.74 | 0.12 | 0.89 |
| *Anaerolineaceae* | 0.80 | 0.78 | 0.73 | 0.33 | 0.28 | 0.91 | 3.11 | 4.01 | 4.48 | 3.87 |
| *Altererythrobacter* | 0.53 | 0.25 | 0.10 | 0.15 | 0.11 | 0.24 | 0.13 | 0.16 | 0.18 | 0.17 |
| *Bacillus* | 0.48 | 3.93 | 0.12 | 0.07 | 0.05 | 0.12 | 0.11 | 0.09 | 0.07 | 0.10 |
| *Arenibacter* | 0.47 | 0.41 | 0.12 | 0.20 | 1.53 | 0.50 | 0.23 | 0.33 | 0.93 | 1.42 |
| *Nocardioides* | 0.39 | 0.26 | 1.17 | 0.73 | 0.43 | 0.70 | 0.58 | 0.47 | 0.74 | 0.56 |
| *Lysobacter* | 0.12 | 0.08 | 1.55 | 3.75 | 2.51 | 1.72 | 0.55 | 1.17 | 0.68 | 0.85 |

WDC, no plant and no inoculant; WDC+M, no plant and inoculant; WDC+P, plant and no inoculant; WDC+M+P, plant and inoculant.

Table.S5 Relative abundance of dominant fungal taxa in waste drill cuttings (WDC). WDC was incubated in a greenhouse for 120 days with and without black locust (*Robinia pseudoacacia*) plant and with or without bacterial and fungal consortium inoculant.

| Taxon | Relative abundance (%) | | | | | | | | | |
| --- | --- | --- | --- | --- | --- | --- | --- | --- | --- | --- |
|  | WDC (Day 0) | WDC+M  (Day 0) | WDC  (Day 60) | WDC+M  (Day 60) | WDC+P  (Day 60) | WDC+M+P  (Day 60) | WDC  (Day 120) | WDC+M  (Day 120) | WDC+P  (Day 120) | WDC+M+P  (Day 120) |
| *Phaeoacremonium* | 5.25 | 38.82 | 2.85 | 44.07 | 6.92 | 37.99 | 5.28 | 35.28 | 25.31 | 3.81 |
| *Microascus* | 0.37 | 7.80 | 0.55 | 17.49 | 0.70 | 16.19 | 2.06 | 29.25 | 22.80 | 1.01 |
| *Acremonium* | 13.29 | 5.83 | 14.67 | 5.30 | 5.56 | 17.12 | 10.41 | 2.19 | 6.12 | 8.41 |
| *Scedosporium* | 0.08 | 9.52 | 0.14 | 5.38 | 0.30 | 6.39 | 0.63 | 3.22 | 1.94 | 0.24 |
| unidentified *Nectriaceae* | 16.62 | 6.89 | 18.50 | 5.16 | 14.15 | 5.00 | 9.49 | 2.10 | 3.13 | 8.77 |
| *Stachybotrys* | 3.97 | 8.10 | 0.55 | 5.39 | 0.87 | 7.96 | 0.84 | 1.89 | 1.42 | 0.29 |
| *Lecanicillium* | 0.01 | 0.00 | 0.00 | 0.00 | 0.00 | 0.00 | 0.00 | 0.00 | 0.15 | 2.94 |
| *Polycephalomyces* | 2.34 | 0.75 | 2.64 | 0.52 | 2.18 | 0.44 | 0.64 | 0.21 | 0.21 | 1.15 |
| *Candida* | 0.00 | 0.00 | 0.04 | 0.03 | 0.01 | 0.03 | 1.20 | 0.50 | 0.05 | 2.64 |
| *Podospora* | 0.00 | 0.00 | 0.04 | 0.02 | 0.03 | 0.09 | 0.08 | 1.42 | 0.05 | 0.40 |

WDC, no plant and no inoculant; WDC+M, no plant and inoculant; WDC+P, plant and no inoculant; WDC+M+P, plant and inoculant.

Table S6 The direct and indirect relationships between variables in the partial least squares path model (PLS-PM) of remediating waste drill cuttings. The path coefficients are calculated by PLS-PM after 1000 bootstraps.

| Relationship | Direct | Indirect | Total |
| --- | --- | --- | --- |
| Microbial inoculant -> Total nitrogen (TN) | 0.362 | 0.000 | 0.362 |
| Microbial inoculant -> Bacterial community | -0.258 | 0.000 | -0.258 |
| Microbial inoculant -> Fungal community | -0.464 | 0.000 | -0.464 |
| Microbial inoculant -> Enzyme activity | 0.632 | 0.000 | 0.632 |
| Microbial inoculant -> Organic matter | -0.537 | -0.145 | -0.682 |
| Black locus -> Total nitrogen (TN) | 0.551 | 0.000 | 0.551 |
| Black locus -> Bacterial community | -0.443 | 0.000 | -0.443 |
| Black locus -> Fungal community | 0.173 | 0.000 | 0.173 |
| Black locus -> Enzyme activity | 0.332 | 0.000 | 0.332 |
| Black locus -> Organic matter | 0.000 | -0.053 | -0.053 |
| Total nitrogen (TN) -> Bacterial community | 0.652 | 0.000 | 0.652 |
| Total nitrogen (TN) -> Fungal community | -0.328 | 0.000 | -0.328 |
| Total nitrogen (TN) -> Enzyme activity | 0.000 | 0.293 | 0.293 |
| Total nitrogen (TN) -> Organic matter | 0.000 | -0.542 | -0.542 |
| Bacterial community -> Enzyme activity | 0.854 | 0.000 | 0.854 |
| Bacterial community -> Organic matter | -0.112 | 0.000 | -0.112 |
| Fungal community -> Enzyme activity | 0.303 | 0.000 | 0.303 |
| Fungal community -> Organic matter | 0.162 | 0.000 | 0.162 |
| Enzyme activity -> Organic matter | -0.814 | 0.000 | -0.814 |
